# The Role of Complement in Organ Transplantation

**DOI:** 10.3389/fimmu.2019.02380

**Published:** 2019-10-04

**Authors:** Monica Grafals, Joshua M. Thurman

**Affiliations:** Department of Medicine, University of Colorado School of Medicine, Aurora, CO, United States

**Keywords:** complement, transplantation, alloimmunity, antibody mediated allograft rejection, delayed graft function, therapeutics

## Abstract

The current immunosuppressive protocols used in transplant recipients have improved short-term outcomes, but long-term allograft failure remains an important clinical problem. Greater understanding of the immunologic mechanisms that cause allograft failure are needed, as well as new treatment strategies for protecting transplanted organs. The complement cascade is an important part of the innate immune system. Studies have shown that complement activation contributes to allograft injury in several clinical settings, including ischemia/reperfusion injury and antibody mediated rejection. Furthermore, the complement system plays critical roles in modulating the responses of T cells and B cells to antigens. Therapeutic complement inhibitors, therefore, may be effective for protecting transplanted organs from several causes of inflammatory injury. Although several anti-complement drugs have shown promise in selected patients, the role of these drugs in transplantation medicine requires further study.

## Introduction

The principal function of the mammalian immune system is to defend the host against infection (1, 2). The immune system consists of two integrated arms—adaptive immunity and innate immunity. The adaptive immune system is primarily comprised of T and B lymphocytes which express highly specific antigen receptors. The diversity of these receptors is generated through somatic gene rearrangement, and T and B cells that express a specific receptor can expand clonally after the cell encounters cognate antigens. Activated T and B cells can also differentiate into memory T cells and B cells, thereby generating long-lived immunological memory of antigens.

Unlike the adaptive system, the innate immune system is comprised of myeloid cells (dendritic cells, monocytes, macrophages, neutrophils), and several other cell types. These cells do not express rearranged receptors, they have limited clonal expansion, and, for the most part, they do not generate memory. Cells of the innate immune system instead express germ-line encoded pattern recognition receptors (PRR) that detect conserved pathogen associated molecular patterns (PAMPs) present in microbes but not shared by healthy mammalian cells (3, 4). The innate immune system also encompasses non-cellular mediators capable of microbial recognition—for example, complement proteins.

Activation of the innate immune system by microbial ligands causes inflammation, the first line of defense against infection, but equally importantly it induces the maturation of antigen-presenting cells (APC) and their migration to secondary lymphoid tissues where they trigger primary T cell and B cell responses. The latter function of the innate immune system is critical for initiating adaptive immunity to infection and vaccines in the naïve host. The innate immune system is therefore responsible for the initial non-self recognition events that ultimately lead to productive T and B cell immunity. It is also generally accepted that innate immunity represents the first step in allograft rejection mechanisms and guides the development of adaptive immune response in transplantation.

Alloimmunity is considered an adaptive immune response, and it represents acquired immunity against foreign antigens that occurs during the lifetime of an individual. Adaptive immunity is antigen specific and reciprocal cognate interactions by T cells play key roles in the generation of alloimmune responses (1–4). Our current armamentarium of immunosuppressive drugs is designed primarily to keep the adaptive immunity in check. However, the role of innate immunity as a significant driver of alloimmune response is increasingly recognized (5–7). The communication between innate and adaptive immunity mainly involves promoting antigen presentation and co-stimulation of cognate B and T cells (7). It is notable, however, that studies of innate immunity after transplantation have most frequently been performed in the context of ischemia-reperfusion (I/R) injury. The activation of innate immunity in the immediate post-transplant period in the context of I/R injury does not fully explain its role in acute rejection, which typically happens weeks to months after transplantation. There is, therefore, an unmet need for the investigation of innate immunity during an episode of acute rejection, especially in human organ transplants (8, 9).

## Overview of the Complement Cascade

The complement cascade is comprised of more than 30 soluble and cell-bound proteins (10). These include PRRs, zymogens that become activating enzymes, biologically active fragments, complement receptors, and complement regulatory proteins. The transplanted organ is exposed to recipient complement proteins as soon as it is reperfused. Conversely, complement proteins and fragments generated within the allograft enter the systemic circulation. Although the complement system is an important effector mechanism for antibody-mediated cytotoxicity, that is only one of its functions. The complement system can be activated in an antibody-independent fashion (discussed below). Complement fragments also modulate T cell differentiation and the B cell response to antigens. Consequently, this system modulates the adaptive immune response, mediates many of the downstream effects of B and T cell immunity, and can function independently of the adaptive immune response. Furthermore, the complement cascade interacts with other biologic systems, including toll-like receptors, the inflammasome, and the clotting cascade (11).

**Figure 1:**
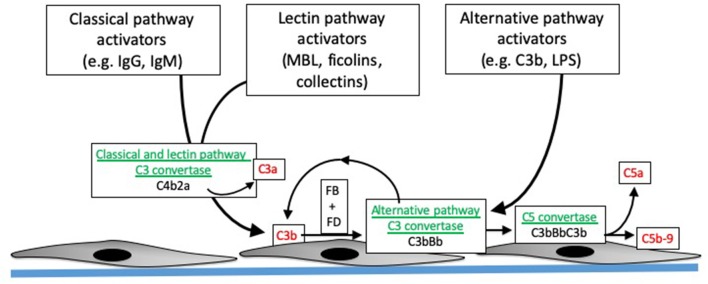
Overview of complement activation. Specific activating molecules engage the classical, lectin, and alternative pathways. Cleavage of C4 and C2 generates C4b2a, the classical and lectin pathway C3 convertases (enzymes that cleave C3). C3 generated by the classical and lectin pathways can combine with factor B (FB), which is then cleaved by factor D (FD), to form C3bBb. C3bBb is the alternative pathway C3 convertase, and can also be generated by spontaneous formation of C3b. The C3 convertases combine with another C3b to form the C5 convertase, which then cleaves C5 into C5b and C5a. C5b combines with C6, C7, C8, and C9 to form C5b-9, or the membrane attack complex. The convertases are depicted in green, and pro-inflammatory molecules generated during complement activation are shown in red.

The complement system is activated through three distinct pathways: the classical pathway (CP), lectin pathway (LP), and alternative pathway (AP). These activation pathways can be engaged by different pathologic processes in the allograft, including donor brain death, I/R injury, and antibody mediated rejection. Although these pathologic processes engage the complement system through distinct molecular mechanisms, the same downstream effectors are generated (Figure 1). The CP is activated by antibodies bound to their target ligands. This may be particularly important in those transplant recipients with donor specific antibodies (DSA) reactive to polymorphic human leukocyte antigens (HLA) expressed on endothelial cells of the allograft.

The LP is activated when activating proteins bind to sugars expressed on the surface of bacteria. LP activation can be initiated by several different proteins, including mannose binding lectin (MBL), collectins-10 and−11 (and maybe collectin-12), and ficolins 1–3 (12). These pattern recognition molecules are complexed with mannose-associated serine proteases (MASPs). When the pattern recognition molecules bind to target ligands, the MASPs become activated and then cleave C4 or, in some cases, activate the alternative pathway (12). The LP is usually activated by binding of these recognition molecules to sugars expressed on bacteria, but they can also bind to ligands expressed on injured cells. Collectin-11, for example, binds to L-fucose expressed on ischemic tubular epithelial cells (13). Cleavage of C4 by either the CP or LP leads to covalent fixation of C4b to nearby surfaces, and the release of the C4a fragment. Genetic variants in the MBL2 gene affect MBL levels, and lower levels of MBL are associated with increased risk of infection. Because the liver is the primary source of MBL, liver transplant recipients who receive organs from donors with MBL2 polymorphisms or mutations can have low MBL levels post-transplantation. Studies have shown that this acquired MBL deficiency is associated with an increased risk of serious infections in the recipient (14, 15).

In contrast to the CP and LP, the AP is continually and non-specifically activated in plasma through a process called “tick-over” (16). Circulating C3 molecules are hydrolyzed, generating a form of C3 [C3(H_2_O)] that can combine with factor B and form a C3 convertase (i.e., an enzyme that cleaves additional C3 molecules). Although C3(H_2_O) cannot bind to surfaces, C3b that is generated by the C3(H_2_O)Bb convertase can covalently bind to nearby surfaces. This C3b can also form convertase (C3bBb), thereby amplifying alternative pathway activation on the target surface. Because tick-over is a spontaneous process, complement regulatory proteins are critical for controlling AP activation. Patients with mutations in the regulatory proteins are, consequently, susceptible to complement mediated diseases, such as atypical hemolytic uremic syndrome (aHUS) and C3 glomerulopathy. Of note, C3b generated by either the CP or LP can also feed into this process. Amplification through the AP may account for the majority of downstream fragment generation, even when complement activation is initiated through the CP (17).

Complement activation through all three pathways leads to cleavage of the C3 protein, generating the C3a and C3b fragments. C3b has a reactive thioester bond that can bind covalently to hydroxyl and amine groups on nearby surfaces, thereby marking, or “opsonizing,” target cells and surfaces. Full complement activation also leads to cleavage of C5, generating soluble C5a and the larger C5b fragment. C5b seeds the formation of the membrane attack complex (MAC, or C5b-9), a multimeric complex that forms a pore in target cells and can cause target cell activation or lysis (18).

### Complement Receptors

Although the MAC directly affects target cells, most of the biologic effects of complement system are mediated by receptors for the various fragments. The C3a receptor (C3aR) and C5a receptors (C5aR1 and C5aR2) are seven-transmembrane receptors that are expressed on myeloid cells and some parenchymal cells. Expression of these receptors can increase in some conditions (19, 20). C5aR1 expression, for example, increases in rejecting murine renal allografts (21). C3aR and C5aR1 are both G-protein coupled receptors, whereas C5aR2 does not have a G-protein coupling motif. It was initially believed to be a decoy receptor that competed with C5aR1 for C5a, although studies have indicated that C5aR2 does have various functions relevant to transplantation, including generation of induced T regulatory cells (22), mediating I/R injury (23), and inhibition of cellular regeneration after ischemia (24).

Complement receptors 2-4 (CR 2-4) are cell-surface receptors that bind to the C3 degradation fragments (iC3b, C3dg, and C3d). CR2 is a transmembrane protein that binds to C3dg and C3d, as well as several non-complement ligands. It is expressed on B cells and follicular dendritic cells, as well as some T cell subsets (25). CR2 signaling lowers the threshold for B cell activation, thereby increasing the B cell response to C3d-opsonized antigens. CR3 and CR4 are β-integrins that can bind to iC3b as well as other ligands (26). CR3 contains an α chain (CD11b) associated with a β2 subunit (CD18). It is expressed on most myeloid cell populations, and it mediates phagocytosis, cell activation, respiratory burst, and cytokine production. It can also negatively regulate the immune response (27). CR4 is comprised of an α unit (CD11c) associated with a β2 subunit (CD18) and is expressed on myeloid cells and some T and B cells. CR4 binds to iC3b (as well as several other ligands) and can increase phagocytosis and cytokine production (28, 29). In spite of all of these immunomodulatory functions, the role of these complement receptors in allograft tolerance and rejection has not been extensively studied.

### Complement Regulatory Proteins

Complement activation is controlled by the specificity of the pattern recognition molecules that initiate activation. Host cells also express several membrane-bound regulatory proteins that negatively regulate activation. These proteins limit complement activation by accelerating the decay of the complement activating complexes (“convertases”), or by inactivating the C3b component of the convertases (30, 31). Several soluble proteins also control complement activation. C1q esterase inhibitor (C1-INH) is the primary inhibitor of the CP and the LP, and C4b-binding protein also controls activation of these pathways. Factor H is an important regulator of the AP. The particular importance of factor H for protecting the body from pathologic AP activation is highlighted by the association of factor H mutations with several diseases (32). Complement-mediated allograft injury indicates that these regulatory proteins can be overwhelmed or subverted in the allograft. Ischemic injury of the kidney, for example, increases local production of activating complement proteins and causes downregulation of regulatory proteins, thereby creating a microenvironment favorable to AP activation (33).

## Pro-Inflammatory Effects of Complement in the Allograft

Once complement is activated within a transplanted organ it can have direct and indirect pathologic effects. As outlined above, multiple different biologically active complement fragments are generated. These proteins and fragments directly affect resident organ cells, they are chemotaxins and activators for neutrophils and macrophages, and they provide important signals for T and B cell activation. The location of complement activation will vary in different settings. In kidney ischemia, for example, activation primarily occurs in the tubulointerstitium (34), whereas in AMR activation occurs in the peritubular capillaries (35). The location of activation determines which cells will be directly affected by MAC or opsonization with C3b. Soluble fragments such as C3a and C5a can have more distant effects, but the site of activation may affect their access to the circulation and peripheral blood cells. It is useful to understand the contribution of the individual complement fragments to injury, as drugs that target specific fragments are in development.

### C3a

Little is known about the specific role of C3a in transplant injury. Nevertheless, studies in mouse models of kidney disease have shown that C3a/C3aR signaling contributes to glomerular and tubular injury (36, 37), and it can promote epithelial to mesenchymal transition (36). C3a also stimulates epithelial cells to produce chemokines which may be an important cause of tissue inflammation (38). There are not currently any specific antagonists of C3a available for clinical use. It is noteworthy, however, that drugs that target complement at the level of C5 will not prevent generation of C3a.

### C5a

C5a has several pro-inflammatory effects and is a potent myeloid cell chemoattractant. C5aR deficiency or blockade is protective in models of I/R injury (39), tubulointerstitial injury (40), and anti-neutrophil cytoplasmic antibody (ANCA) vasculitis (41). In a murine kidney transplant model, a small molecule C5aR1 antagonist prolonged the survival of mismatched allografts (21). The agent reduced infiltration by monocytes/macrophages, and also decreased priming of T cells in the recipients. C5aR antagonists have been developed for clinical use.

### Membrane Attack Complex

The formation of sublytic MAC on endothelial cells leads to NF- κb activation within the cells (42–44), inducing the cells to produce IL-1α and IL-8 (45). In a heart transplant model, this effect was also associated with activation of allogeneic CD4 T cells (44).

## The Role of Complement in the Adaptive Immune Response

Although complement activation can cause direct inflammatory injury of the allograft, it can also enhance the response of B and T cells to donor antigens. Signaling through CR2 increases the B cell response to T-dependent antigens (46). Thus, B cells have a stronger response to antigens that are opsonized with C3d. Complement activation in tissues after I/R amplifies generation of antibodies to foreign antigens, although it is not clear whether this is a specific effect of complement on the B cell response or whether it is indirectly caused by cytokines generated downstream of complement-mediated injury (47). Several studies have linked complement activation in transplanted organs with the development of T cell alloreactivity (21, 48). This may be due to a co-stimulatory effect of complement fragments, but experiments have also shown that C3a and C5a reduce the inhibitory function of T regulatory cells (49). Elegant work has also shown that complement proteins produced by dendritic cells and T cells are activated at the cell-cell interface, and enhance the T cell response to antigen (50). Although complement inhibitors would likely block this mechanism of T cell activation, it is not driven by complement activation within the allograft *per se*.

In some settings, complement activation can also limit the adaptive immune response. Studies in several cancer models have shown that complement activation within tumors can attract myeloid derived suppressor cells (MDSCs) that block T cell anti-tumor immunity (51, 52). Although there are many differences between tumor immunology and transplant immunology, some parallels have been seen. For example, co-stimulatory blockade can induce allograft tolerance in murine models. In this setting, C5aR deficiency reduced infiltration of heart allografts by a myeloid suppressor cell population that is necessary to maintain tolerance, similar of the effect in tumors (53). Another mechanism of complement-mediated immunosuppression has also been identified in the liver. Stellate cells produce iC3b which, in turn, causes dendritic cells to differentiate into MDSCs (54). This finding may explain why greater tolerance is seen in recipients of liver transplants that contain the stellate cells than transplants containing isolated hepatocytes.

### Complement in Ischemia/Reperfusion Injury

Numerous studies have shown that complement is activated after I/R, although the mechanisms may vary between different organs. Complement activation in the ischemic heart and intestine may be initiated by immunoglobulin, but it primarily involves the LP (55, 56). In the kidney, complement activation primarily involves the AP and does not require immunoglobulin (34, 57, 58). Studies in which kidneys from C3 deficient mice were transplanted into wild-type recipients revealed that the allograft itself may be an important source of complement proteins involved in tubulointerstitial activation (48, 59).

Complement inhibitory drugs have proven effective in several pre-clinical models of I/R injury. An inhibitory antibody to C5 (which prevents formation of C5a and the MAC) and a small molecule C5a receptor antagonist were each protective in models of cardiac (60, 61) and kidney (39, 62) I/R injury. LP blockade was protective in models of kidney and cardiac I/R injury (56). A monoclonal antibody that inhibits the AP was protective in a model of warm I/R injury of the kidney (58). This same drug also prevented I/R injury in a mouse kidney transplant model, and it also reduced T cell mediated rejection of the organs (63).

In spite of promising pre-clinical data, a trial that enrolled 27 kidney transplant patients at high risk of DGF who were randomized to treatment with an inhibitory monoclonal antibody to C5 (eculizumab) did not show any benefit with treatment (64). Another clinical trial is ongoing, however, in which kidneys treated with an agent that attaches a complement regulator to cell membranes (65). This approach was previously shown to be beneficial in a rat kidney transplant model (66).

## Antibody Mediated Rejection

**Figure 2:**
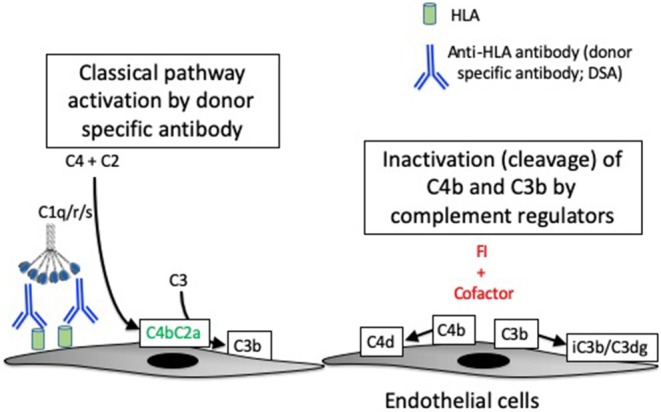
Complement activation in antibody-mediated rejection. Antibody-mediated rejection is caused by binding of antibodies to human leukocyte antigens (HLA) expressed on endothelial cells of the transplanted organ. The antibodies (referred to as donor specific antibodies, or DSA) activate the classical pathway of complement. Classical pathway activation causes the cleavage of C4, and one of the resultant C4 fragments (C4b) is covalently attached to target surfaces. C4b comprises part of the classical pathway C3 convertase, C4b2a. C3b can become covalently attached to target cells, similar to C4b. A protease called factor I (FI) controls complement activation by cleaving the C4b and C3b molecules, thereby stopping convertase activity. Although they are no longer catalytically active, the C4d and C3dg fragments remain bound to the target cells and can be detected by immunostaining of tissue biopsies.

In patients with acute and chronic AMR, DSA binds to donor HLA expressed on endothelial cells and activates the CP. Complement activation on the endothelial cell surface is believed to be an important cause of injury to the capillaries (Figure 2) (35, 67). The diagnosis of AMR is based on detecting DSA in the plasma, microvascular inflammation on a biopsy (e.g., glomerulitis or peritubular capillaritis in allografts), and C4d deposition in the peritubular capillaries (68). However, the diagnostic criteria have been modified to account for C4d-negative cases (69, 70). It is not known whether the C4d-negative cases of AMR are caused by non-complement-mediated injurious effects of the DSA, or whether it simply reflects variability in the ability to detect the C4d.

Because complement activation by the DSA is such an important component of AMR, assays have been developed to distinguish the complement activating potential of DSA in the circulation. These assays identify immunoglobulin that binds to specific HLA types, and also tests whether the detected antibodies bind to C1q (71) or carry a C3d molecule (72). Patients with DSA that bind to C1q or to which C3d is bound are at greater risk of developing AMR and they have a worse overall prognosis (73). These findings highlight the importance of the complement system in AMR, and potentially provide a test for identifying patients at risk of AMR. An inhibitory antibody to C5 was protective in a model of heart transplantation in highly sensitized mice, supporting the importance of complement activation in the pathogenesis of microvascular injury (74). Interestingly, treatment with the C5 inhibitor led to long term allograft survival, even though DSA persisted after the treatment was stopped. It is possible that complement inhibition induced “accommodation” in the allograft. Accomodation is a state in which an allograft becomes resistant to AMR. This may occur through upregulation of complement regulatory proteins, altered expression of the target antigens by the allograft, or changes in the isotype of the DSA (75).

Eculizumab has been used in transplant recipients at high risk of developing AMR, as well as patients with active disease refractory to treatment. Positive results have been reported in lung and kidney transplant recipients with AMR (76–78). Larger series in transplant patients have not shown a consistent benefit, however, and the role of eculizumab for preventing or treatment AMR is not yet clear (73, 79, 80). C1-INH is a substrate-like serine protease inhibitor that blocks several proteases, including C1r, C1s, and the MASPs (81). Originally used as a replacement therapy for patients with hereditary angioedema who have deficiency of C1-INH protein, it has also been tested as a treatment of AMR (82). C1-INH appeared to be beneficial in a small trial of six AMR patients who were refractory to conventional therapy (83), and a larger clinical trial is currently underway.

A clinical trial in patients with ANCA-associated vasculitis has shown that a C5aR1 antagonist is beneficial and may reduce the need for corticosteroids in this disease (84). Although the drug has not yet been approved for this indication, the study demonstrated that it can safely be used in patients with kidney disease. Given that there is pre-clinical data showing that C5aR1 blockade may be a beneficial treatment for rejection (21), this approach holds promise as a novel treatment for transplant patients.

### Xenotransplantation

The critical shortage of human donor organs limits the number of allotransplants, and there has been a long-standing interest in xenotransplantation as a means of increasing the number of available organs. One of the major obstacles to xenotransplantation is hyperacute rejection of the transplanted organ due to natural antibodies (85). Mammals have a pre-existing repertoire of natural antibodies reactive against several sugar motifs, including anti-Galα1,3Gal (86), which is expressed on pig endothelial cells. Natural antibodies bind these endothelial antigens almost as soon as the xenograft is reperfused and lead to hyperacute rejection. Strategies for preventing this process include deletion of the α1,3-galactosyltransferase gene in the donor animal (87), or transgenic expression of human complement regulatory proteins in the allografts (88). Complement inhibitory drugs may be beneficial in this setting, but they would likely need to be administered long-term as the pathogenic natural antibodies may persist in spite of immunosuppression.

## T Cell Mediated Rejection

As outlined above, complement deficiency and/or inhibition can reduce alloreactivity to allografts. Complement-mediated T cell priming may occur at the T-cell/dendritic cell interface, or it may occur downstream of complement activation within the allograft. For example, antibody-induced complement activation on allograft endothelial cells can promote activation of T cells (44).

An interesting discovery was that local production of complement proteins increases after ischemia, and that expression of these proteins by the allograft is associated with T cell mediated rejection (48). Transplantation is a unique setting that allows distinction of local complement production in the allograft from hepatic production in the recipient, as donor C3 may be of a different allotype than recipient C3. C3 allotypes have been defined as fast (F) and slow (S) based on a polymorphism that affects the mobility of the protein on electrophoresis. One study of patients who expressed a different C3 allotype than the allograft they received, reported that the percentage of plasma C3 generated in transplanted kidneys increases during acute rejection episodes (89). Furthermore, C3 generation in organs from brain dead donors may already be increased at the time the organs are harvested, possibly adversely affecting the survival of these organs (90, 91). Interestingly, the C3 allotype expressed by the allograft may affect the long-term prognosis. Patients expressing the C3S allotype had better outcomes if they received allografts that expressed C3F (either C3F/S or C3F/F) (92).

## Recurrence of Primary Disease in the Allograft

Most forms of primary glomerulonephritis recur in allografts in spite of immunosuppression. Although the drugs usually employed to protect the allograft may reduce the generation of autoantibodies, they probably do not have much effect on production of complement proteins by the liver or activation of complement by immune complexes. Consequently, if a disease like membranous nephropathy recurs after transplantation, complement activation by deposited immune-complexes will have the same effect that it has in disease of native organ.

C3G and aHUS, two glomerulopathies caused by uncontrolled AP activation, are particularly likely to recur in the transplanted kidney. C3G is among the causes of primary GN with the highest rates of recurrence (93–95). Atypical HUS also frequently recurs in renal transplant patients, particularly in patients with factor H mutations. In a recent case series, 16 of 19 patients had disease recurrence within 7 years of transplantation in spite of treatment with standard immunosuppression (96), and recurrence of aHUS is particularly high in the peri-transplant period (97). This may be due to the inability of these patients, many of whom have molecular defects in AP regulation, to resolve ischemia-induced AP activation in the allograft.

## Complement Biomarkers

During complement activation, complement protein fragments are released into the plasma, and C3 and C4 fragments are covalently fixed to target tissues. Native kidney biopsies are routinely stained for C3 deposits, and in some centers they are also stained for C4 fragments (98). Because C4 is covalently attached to target tissues, C4 deposits provide a durable marker of CP activation. Allograft biopsies are now routinely stained for C4d, and detection of C4d in the peritubular capillaries is interpreted as a marker of classical pathway activation in patients with AMR (99). CP activation on the capillary would also be expected to result in C3 fragment deposition, although C3d deposition seems to be a less sensitive indicator of AMR (100). It is possible that C3d deposition signifies more complete activation of the complement cascade, and one study found that deposition of C3d on the peritubular capillaries was associated with a worse prognosis (101). In contrast to AMR, I/R injury of native kidneys is associated with C3d deposition on the tubules in the absence of C4d, consistent with AP activation at this location (57). Thus, distinct patterns of complement activation may be useful for identifying the underlying cause of organ injury.

Soluble complement fragments can be measured in body fluids by enzyme linked immunosorbent assays (ELISAs). The half-life of these fragments is short, so elevated levels of complement fragments indicates that there is ongoing activation (102). There are assays that can measure many different complement fragments, including C4a, C3a, Ba, Bb, C5a, and soluble sC5b-9. Measurement of these fragments, therefore, can also shed light on the underlying pathologic process. It was recently reported, for example, that Ba fragments are elevated in the urine of patients with ischemic acute kidney injury, indicating that the AP is activated in these patients (103). C4a levels are increased in patients with severe SLE, on the other hand, indicating activation of the CP in this immune-complex disease (104). Other than staining allograft biopsies for C4d, complement biomarkers are not routinely analyzed in transplant recipients. As the use of complement inhibitory drugs expands, however, there will be an increasing need to develop accurate biomarkers.

## Complement Therapeutics

As described above, studies have tested whether eculizumab is useful for preventing complement activation in the allograft caused by ischemia and AMR. It has also been used in transplant recipients with post-transplant aHUS recurrences (105). C1-INH has also been used to prevent AMR, and it is being tested for treatment of the disease in an ongoing clinical trial. Pre-clinical work has shown that other complement inhibitory agents may be useful in the transplant setting, including an AP inhibitor (63), a LP inhibitor (56), and C5a blockade (21). Many new complement inhibitory drugs are in clinical development, some of which will likely soon become available for clinical use (106, 107). In particular, a C5a inhibitor has shown some efficacy in patients with ANCA associated vasculitis (84).

## Future Directions

Although immunosuppressive medications have improved short term transplant outcomes, long-term outcomes have not shown an equivalent improvement. Events at the time of transplantation can affect long-term outcomes, including brain death of the donor and I/R injury of the allograft. Acute and chronic AMR are also important causes of allograft failure, and currently there are no specific therapies shown to be effective for AMR. Complement activation may contribute to all of these forms of injury. Furthermore, complement activation provides important signals that enhance the adaptive immune response, thus linking inflammation in allografts with long-term alloimmunity. The standard immunosuppressive medications used to prevent transplant rejection do not directly block the complement cascade. Thus, complement inhibitory drugs may be useful adjuncts to the currently available anti-rejection drugs in several different clinical settings.

Although eculizumab and C1-INH have shown promise in case reports and small series, their role in transplant medicine requires further study. Many additional anti-complement therapeutics are in clinical development, and some of these new drugs block individual activation pathways or specific components of the complement cascade. This could potentially allow clinicians to block the parts of the complement cascade involved in allograft injury while leaving other parts of the cascade active.

However, testing these new agents in the transplant setting poses several challenges. First, all transplant recipients are treated with multiple immunosuppressive drugs. Thus, new drugs will need to be tested as add-on treatments to these other agents. Second, even within a single diagnosis, such as AMR, there is patient heterogeneity. Complement activation may not be an important part of C4d-negative AMR, for example. The development of new complement biomarkers may therefore be critical for selecting patients most likely to benefit from complement inhibitors, and for discerning a response to treatment.

## Author Contributions

All authors listed have made a substantial, direct and intellectual contribution to the work, and approved it for publication.

### Conflict of Interest

JT receives royalties from Alexion Pharmaceuticals, Inc. JT is also a consultant for AdMIRx, Inc., a company developing complement inhibitors. He holds stocks and will receive royalty income from AdMIRx. The remaining author declares that the research was conducted in the absence of any commercial or financial relationships that could be construed as a potential conflict of interest.
